# Roles of sliding-induced defects and dissociated water molecules on low friction of graphene

**DOI:** 10.1038/s41598-017-17971-1

**Published:** 2018-01-09

**Authors:** Zaixiu Yang, Sukanta Bhowmick, Fatih G. Sen, Anindya Banerji, Ahmet T. Alpas

**Affiliations:** 1 Engineering Materials Program, Mechanical, Automotive and Materials Engineering Department, University of Windsor, Windsor, Ontario, N9B 3P4 Canada; 2 Center for Nanoscale Materials, Argonne National Laboratory, Cass Ave, Lemont, IL 60439 USA

## Abstract

Sliding contact experiments and first-principles calculations were performed to elucidate the roles of structural defects and water dissociative adsorption process on the tribo-chemical mechanisms responsible for low friction of graphene. Sliding friction tests conducted in ambient air and under a dry N_2_ atmosphere showed that in both cases a high running-in coefficient of friction (COF) occurred initially but a low steady-state COF was reached only when the sliding was continued in air with moisture. Density functional theory (DFT) calculations indicated that the energy barrier (*E*_*b*_) for dissociative adsorption of H_2_O was significantly lower in case of reconstructed graphene with a monovacancy compared to pristine graphene. Cross-sectional transmission electron microscopy of graphene transferred to the counterface revealed a partly amorphous structure incorporating damaged graphene layers with d-spacings larger than that of the original layers. DFT calculations on the reconstructed bilayer AB graphene systems revealed an increase of d-spacing due to the chemisorption of H, O, and OH at the vacancy sites and a reduction in the interlayer binding energy (*E*_*B*_) between the bilayer graphene interfaces compared to pristine graphene. Thus, sliding induced defects facilitated dissociative adsorption of water molecules and reduced COF of graphene for sliding tests under ambient and humid environments but not under an inert atmosphere.

## Introduction

The reduction of friction by using carbon-based materials either in the bulk form, or as surface coatings, is an important technological alternative to the usage of liquid lubricants^1–3^. The low coefficient of friction (COF) of graphite arises from shearing of the basal planes but the COF is not an intrinsic property^4^ as it depends on the presence of dissociated water and/or oxygen molecules in the environment^5–8^. Similarly, low COF values of polycrystalline diamond (PCD) and diamond-like carbon (DLC) coatings observed under the ambient conditions were often attributed to the saturation of the dangling carbon bonds at the contact surfaces by dissociatively adsorbed gas molecules^9–11^. Ideally, graphene is a two dimensional array of carbon atoms, characterized by Raman peaks at 1580 cm^−1^ and 2700 cm^−1^ ^12^. While the 2D peak of graphite at 2700 cm^−1^ consists of two separate components, namely 2D^1^ and 2D^2^, a single sharp 2D peak is the distinguishing feature of graphene^13^. Low-load (1 nN) sliding experiments conducted using friction force microscope showed that the multilayered graphene with more than four layers (produced by mechanical exfoliation) exhibited a COF of 0.2 when tested under an ambient atmosphere with a relative humidity (RH) of 25% compared to a higher COF of 0.4 for the monolayer graphene^14^. Ethanol processed monolayer graphene tested using a ball-on-disk tribometer at a load of 1N under a dry H_2_ atmosphere showed a low COF of 0.22, whereas once the same sliding tests were conducted on three or four-layer graphene the COF decreased to 0.15^15^. CVD deposited multilayer graphene tested using a pin-on-disk tribometer at 1N showed a high COF of 0.52 in a dry N_2_ atmosphere, but exhibited a low steady state COF value of 0.11 in an air atmosphere with 45% RH^16^.The computational studies based on density functional theory (DFT) showed that hydroxyl groups on pristine graphene could reduce the adhesion between graphene layers contributing to low friction^17^, although the relation between adhesion and friction should be interpreted with caution, as it has been suggested that the friction force would depend on other factors such as adhesion energy hysteresis^18^.

Considering the friction behaviour of other carbon based materials, polycrystalline diamond (PCD) tested in an ambient air with 37% RH against a ceramic counterface was shown to exhibit a COF of 0.10, but a COF of 0.55 was recorded in a dry N_2_^19^. A high COF of 0.70 for nanocrystalline diamond sliding in high vacuum (10^−9^ Pa) was reported in another study, which also observed that introduction of water vapour reduced the COF to <0.05 while in a dry H_2_ atmosphere COF values <0.01were recorded^20^. The low COF of nanocrystalline diamond in water vapour and hydrogen atmospheres were attributed to OH and H termination of surface C atoms. The non-hydrogenated grades of diamond-like carbon (NH-DLC) coatings showed a reduction in COF with an increase in the humidity^10^. It was proposed that the dangling sp^3^ carbon bonds in PCD were terminated by a physisorbed water layer while for the NH-DLC the sp^2^ hybridized carbon atoms were converted to sp^3^ as a result of moisture adsorption^11,21^.

Density functional theory (DFT) based calculations showed that the chemisorption of C atoms by H atoms on a diamond (111) surface would reduce the work of separation between an Al interface and H terminated C atoms to 0.02 J/m^2^ compared to 4.08 J/m^2^ for C without H termination^22^. For a diamond (111) surface, DFT computations predicted a low H_2_O dissociation energy barrier (*E*_*b*_) of 0.122 eV^23^. Contrary to diamond, pristine graphene does not possess dangling bonds. According to DFT calculations a large *E*_*b*_ is required for dissociative adsorption of H_2_O on pristine graphene^24^.

Formation of sliding-induced defects should be considered as an important part of friction mechanisms of graphene. Raman spectroscopy of the worn graphene surfaces indicated generation of D, and split G peaks due to the formation of defects during sliding contact^16^. Edge fracture of graphene plates within the wear tracks were also reported^25^. Static atomic force indentation experiments revealed a reduced elastic modulus for graphene upon the introduction of vacancies^26^. A decrease in the fracture strength of graphene was reported to occur as a result of introduction of Ar^+^ irradiation defects^27^. It was shown that vacancy formation and adsorption of the hydroxyl groups would reduce the strength and elastic modulus of graphene^28^.

H_2_O dissociation processes may be facilitated by the formation of defects in graphene^29^. Comparision of *E*_*b*_ values calculated for water dissociation on graphene using different computation techniques indicated that water dissociation process favoured the monovacancy graphene but not the pristine graphene^24,30,31^. Among various types of defects that may exist in graphene Stone-Wales defects were shown to have lower formation energy than a monovacancy^32^, and since C atoms at these defects do not possess dangling bonds, the binding of OH- or H- groups to the C atoms would be less likely. The reactivity of defective graphene with H, F and phenyl groups was reported to decrease in the following order; monovacancy > hydrogenated zigzag edge > double vacancy > Stone-Wales > hydrogenated armchair edge > defect-free graphene^33^. Monovacancy graphene (MG) was frequently used to simulate the dissociation mechanisms of molecules like H_2_O and O_2_^24,30,31,34–36^, and different *E*_*b*_ values were reported depending on the simulation methods (potentials, cell sizes) used. An *E*_*b*_ of 0.04 eV for H_2_O dissociation was found by means of DFT calculations without non-local dispersion terms using a cell size of 3 × 3^24^, but a higher value was calculated using a larger cell size of 4 × 4^30^.

The current manuscript elucidates for the first time the friction reduction mechanisms in graphene by considering the roles of dissociated water molecules on the sliding induced defect sites in graphene. The research unraveled the significant roles of sliding-induced graphene defects and transfer layers generated on the counterface and water dissociation in reducing the friction. Using atomistic calculations, and sliding friction experiments it was shown that dissociated water molecules can reduce the graphene interlayer biding energy and may increase the interlayer distance between the adjacent layers as observed by analytical high resolution microscopy techniques. First-principles calculations with vdW functional^37^ were used to model the interfacial tribo-chemical mechanisms. The effects of dissociative adsorption of water molecules between the graphene layers proved to be an important factor in rationalizing the friction reduction mechanisms of graphene.

## Results and Discussion

### Frictional behaviour and observations of worn surfaces

**Figure 1:**
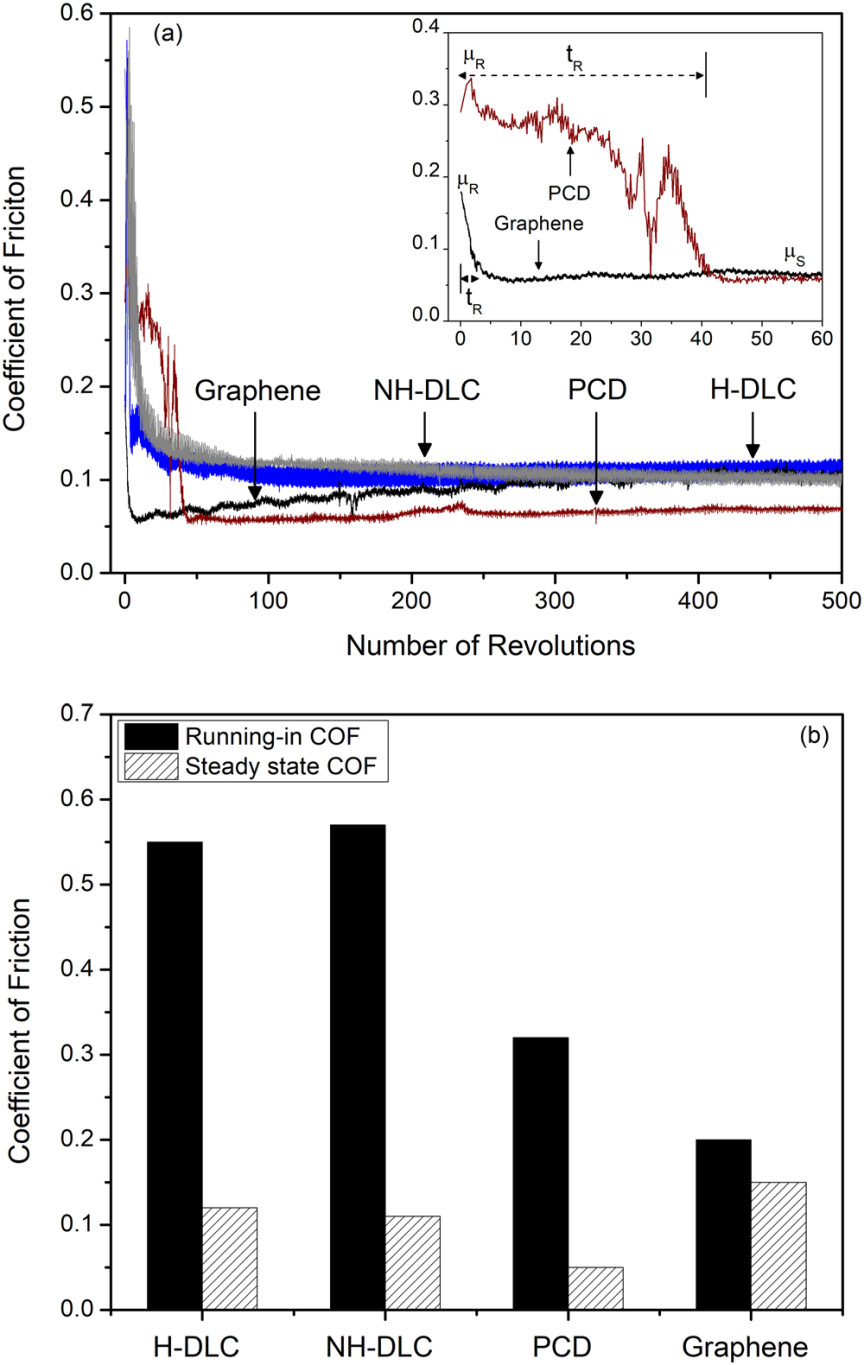
(**a**) The variation of COF of graphene as a function of the number of revolutions (22% RH). The variations of COF of PCD, H-DLC and NH-DLC against the same Ti-6Al-4V counterface are also shown (22% RH). The inset shows the enlarged view of the COF curves of graphene and PCD for the first 60 cycles where µ_R_, t_R_ and initial stages of µ_s_ are marked. (**b**) Average values of running-in (µ_R_) and steady state coefficient of friction (µ_S_) of graphene, H-DLC, NH-DLC and PCD against Ti-6Al-4V, where the μ_R_ and μ_S_ values are the average of three tests.

The variation of the COF of graphene with the number of revolutions for sliding contact tests conducted under an atmosphere with 22% RH is shown in Fig. 1a along with the variation of COF values of PCD and two types of DLCs. The friction curves consisted of an initial running in period (t_R_) where the COF values reached a peak followed by a steady state period. The graphene provided a low running in COF, µ_R_ and the transition to the steady state regime was immediate. The average steady state COF was low at µ_s_ = 0.10 for this sample. The curves shown in Fig. 1a represent the typical friction trends for each type of materials tested, and the results of average µ_R_ and µ_s_ values obtained from all friction tests are presented in Fig. 1b. The average µ_R_ for graphene was 0.20, with a t_R_ of 1–2 revolutions, and the average µ_s_ was 0.15. DLC coatings exhibited high µ_R_ values_,_ i.e. 0.55 for hydrogenated diamond-like carbon (H-DLC) and 0.57 for NH-DLC, and t_R_ for the DLCs were short, t_R_ <10 revolutions. The µ_s_ values recorded for the both DLC coatings were 0.10. The PCD showed the longest t_R_ of 40 revolutions (see Fig. 1a) and a µ_R_ of 0.32. The µ_s_ recorded for PCD was the lowest at 0.05 compared to other samples. Thus, the results suggested that all materials exhibited similar µ_s_, however, graphene showed the lowest µ_R_ and the shortest t_R_. An observation common to all tribological systems was the formation of transfer layers on the counterfaces during the running-in period.

**Figure 2:**
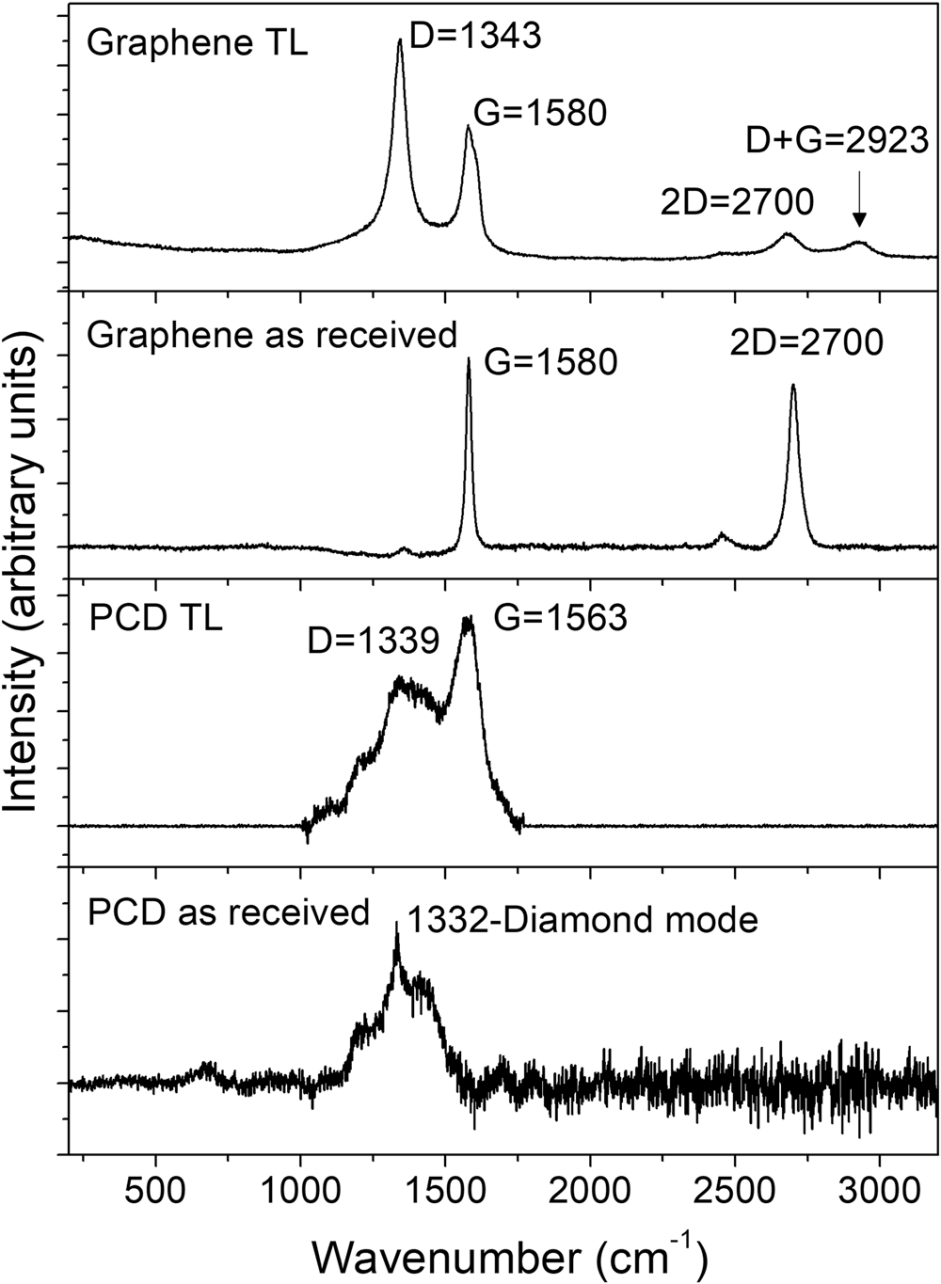
Raman spectra of transfer layers (TL) formed on the counterfaces after sliding tests (22% RH) conducted on graphene and PCD. Raman spectra of as received graphene and PCD prior to the sliding tests are also plotted for comparison.

The Raman spectra of the graphene transfer layer (Fig. 2) revealed formation of a prominent D peak at 1343 cm^−1^ along with a small intensity (D + G) peak at 2923 cm^−1^. Neither of them was present in spectra of pristine graphene. A reduction in the intensity of the 2D peak (2700 cm^−1^) and formation of a split of G peak (1580–1600 cm^−1^) were also observed^12,38^. These results suggest that sliding contact altered the structure of pristine graphene by increasing the degree of disorder in the graphene network. A G peak appeared at 1563 cm^−1^ in the transfer layers of PCD (see Fig. 2), revealing the possibility of surface graphitization during sliding contact^12,21,39,40^.

**Figure 3:**
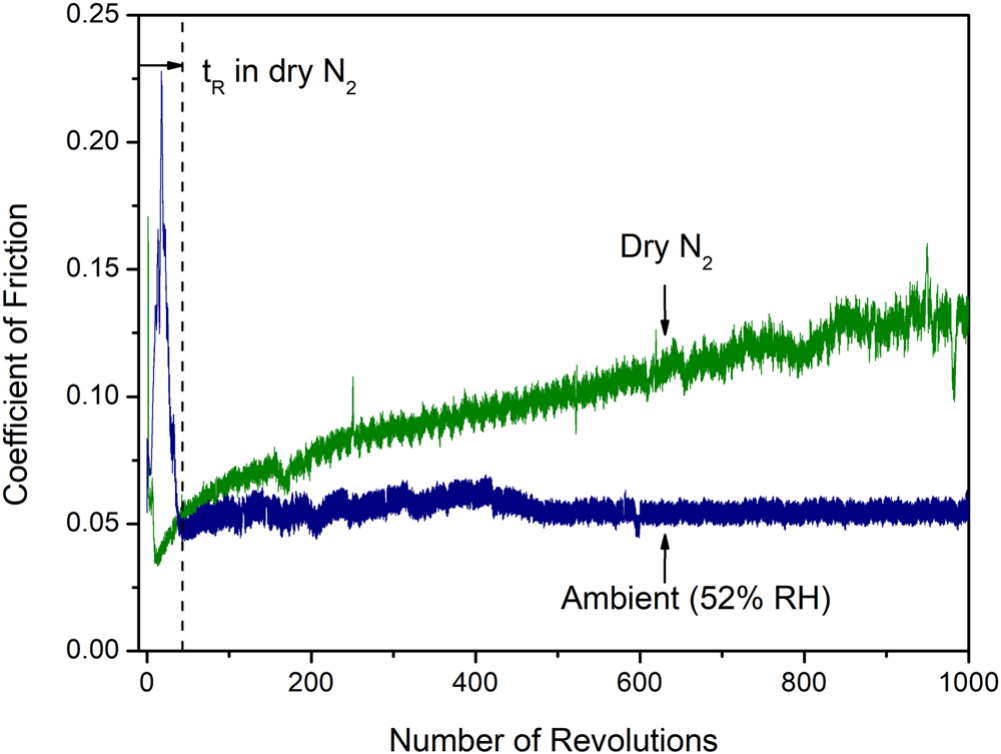
Variation of COF of graphene as a function of number of revolutions when sliding under a dry N_2_ atmosphere against a H-DLC counterface, and when the test was initiated in dry a N_2_ atmosphere and continued in air (52% RH) after 50 revolutions.

During sliding of the graphene, easy formation of transfer layers does not automatically lend itself to a low COF, and the moisture in environment plays an important role in this process. As the above experiments were carried out at a constant humidity of 22% RH, a new set of experiments were conducted to elucidate the role of the transition between the inert-to-humid atmosphere on the frictional properties of graphene. The effect of humidity on controlling the friction of the graphene can be readily understood by considering the two experiments shown in Fig. 3. Both sliding experiments were initiated in a dry N_2_ atmosphere (<4% RH), and a H-DLC counterface was selected to prevent metallic material transfer that would otherwise occur to the graphene surface under dry and inert atmospheres^25^. At the beginning of both experiments, a µ_R_ peak was observed during the running-in period where initial surface damage occurred and once the transfer layers were formed on the counterface the COF was reduced. For the sliding experiments that were continued to be carried out in a dry N_2_ the COF continuously increased and reached 0.14 after 1,000 revolutions, no steady state behaviour could be observed. However, when the chamber was purged with air with 52% RH at the end of the running-in period, a low and steady state COF ensued with µ_s_ = 0.05.

### Dissociative adsorption of H_2_O on graphene

**Figure 4:**
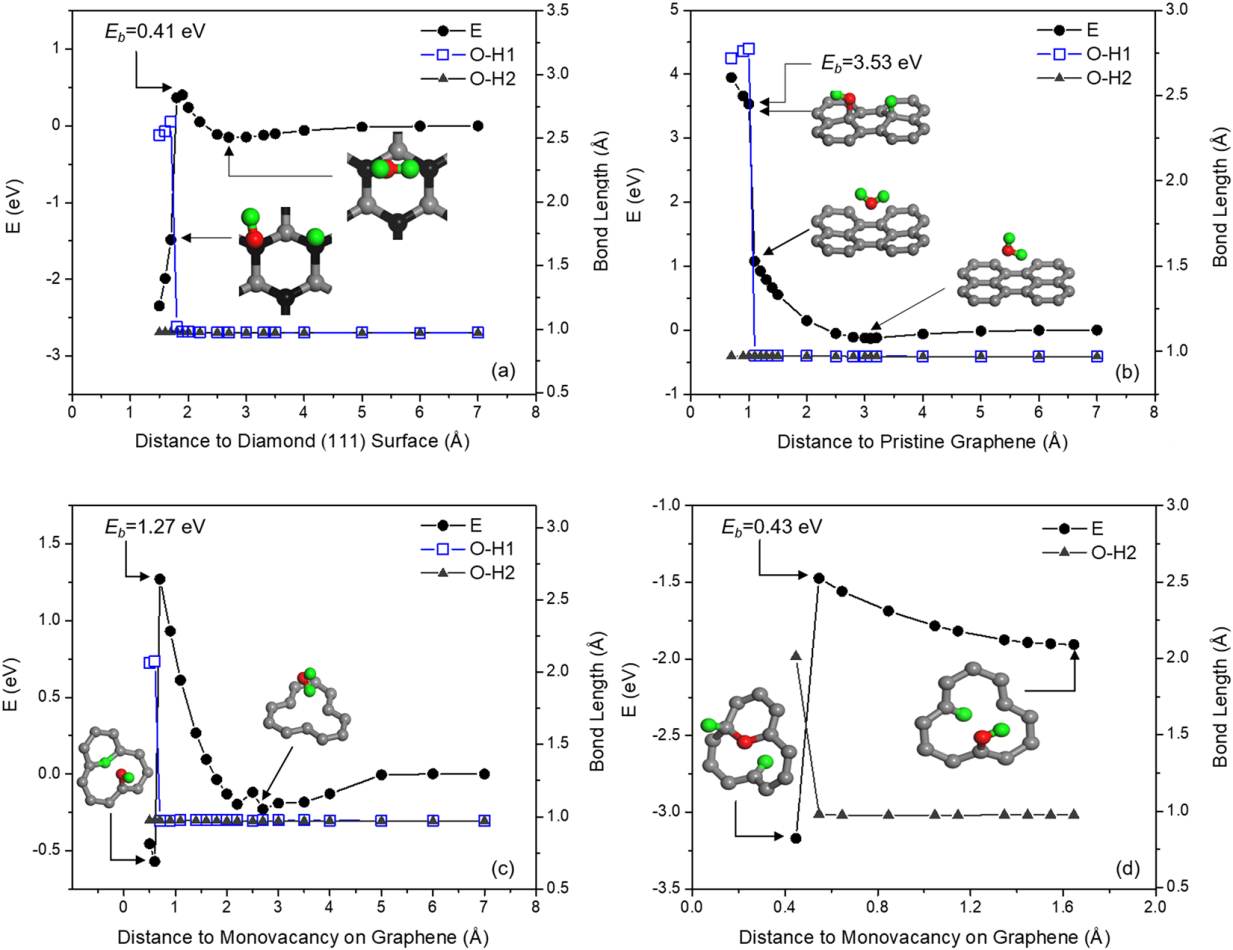
The change of energy and the O-H bond lengths when a H_2_O molecule approached (**a**) the diamond (111) surface; (**b**) the pristine graphene surface; (**c**) the monovacancy graphene surface. (**d**) The change of energy and O-H2 bond length when the OH molecule approached monovacancy graphene surface.

The thermodynamics and structural aspects of interactions between graphene surfaces and H_2_O molecules were investigated using spin-polarized density functional theory (DFT) calculations. The details are given in the ‘Methods’ section. Starting with diamond structure as a reference, Fig. 4(a) shows the energy changes and the variations of bond lengths of O-H1 and O-H2 as a water molecule approaches to diamond surface. When the water molecule approached the diamond surface, O-H1 bond length increased to 2.63 Å at the distance of 1.7 Å and the bond was broken while the bond length of O-H2 stayed unchanged, as indicated in Fig. 4(a). *E*_*b*_ for water dissociation at the bridge site of diamond (111) surface was low at 0.41 eV. The total energies and changes in the O-H1 and O-H2 bond lengths of an H_2_O molecule as it approaches the surfaces of pristine graphene (PG) and monovacancy graphene (MG) are plotted in Fig. 4(b) and (c). When the H_2_O molecule approached the PG surface, the energy of the system progressively decreased and dropped to a minimum of −0.13 eV at a H_2_O-PG distance of 3.1 Å with no change in the O-H bond length as shown in Fig. 4(b). This state corresponds to the physisorption of the water molecule on PG for which the van der Waals interactions were taken into account in the calculations. The adsorption energy of water molecule on PG is therefore −0.13 eV, which is in agreement with the energy values of −0.102 ~ −0.135 eV calculated by coupled cluster singles and doubles (CCSD) correlation method^41^. The energy then increased to 1.08 eV due to repulsion between H_2_O and graphene when the water molecule brought to 1.1 Å away from the PG surface with no change to the O-H bond length, but during this process graphene became subjected to out-of-plane deformation. At a distance of 1.0 Å, the O-H1 bond length increased abruptly to 2.77 Å while almost no change in the O-H2 bond length occurred, indicating that the H_2_O molecule was dissociated into -OH and -H and chemisorbed on the PG surface. The energy of the system increased to 3.53 eV once the water was dissociated. Thus, this process is endothermic and due to the inert nature of PG the energy barrier *E*_*b*_ for dissociative adsorption of water molecule is high. Here, *E*_*b*_ = 3.53 eV is defined as the energy difference between the maximum energy before water dissociation and the energy of the reference state. Using DFT calculations that employed B3LYP:DFTB-D^42^ an *E*_*b*_ of 4.4 eV was previously reported for water dissociating on graphite (per molecule) and the use of GGA/Perdew-Wang 91 provided an *E*_*b*_ of 3.64 eV^24^.

For a water molecule approaching the MG surface (Fig. 4c), the energy decreased to 0.23 eV at the separation distance of 2.7 Å and no change in either of the O-H bond lengths was observed when the water molecule was physisorbed at the vacancy site of graphene. Figure 4(c) also shows that when the water molecule was brought to a distance of 0.7 Å from the MG surface, an *E*_*b*_ of 1.27 eV was observed for water dissociation. At this distance range, the water molecule reoriented itself above the vacancy site, but the O-H bond length did not change. However, at the separation distance of 0.6 Å, the bond length of O-H1 increased to 2.20 Å, indicating again that water molecule was dissociatively adsorbed at the vacancy site of graphene with formation of C-OH and C-H. Meanwhile the bond length of O-H2 remained unchanged. The *E*_*b*_ for water dissociation on MG was lower compared to the *E*_*b*_ for water dissociation on PG Fig. 4(b), for which additional energy was required for sp^2^ to sp^3^ transformation. It is also observed in Fig. 4(c) that due to the dissociative adsorption of water molecule the energy dropped by 1.84 eV to −0.57 eV, indicating that this process was spontaneous.

The structure of MG with O-H2 and H1 chemisorbed on its surface was in a metastable state relative to the structure of completely dissociated water. The changes in the energy and the O-H2 bond length with the distance between O-H2 and MG are plotted in Fig. 4(d). The energy increased by 0.43 eV at 0.5 Å followed by a reduction to −3.17 eV corresponding to an increase in O-H2 bond length to 2.0 Å, indicating breaking of the O-H2 bond. As a result of this process all the three dangling bonds at the vacancy site became saturated by the two H atoms and the O atom by overcoming an *E*_*b*_ of 0.43 eV. Since the water dissociated structure in Fig. 4c shows that one H atom was attached to two dangling C atoms, and the OH was attached to the other dangling bond at the monovacancy site, there is no available dangling C bond left to assist with further dissociation of OH. However, the structure in Fig. 4c represents a metastable state; Water should be completely dissociated at the monovacancy site to reach the lowest energy state as reported in the literature^24,30^. Therefore, the structure needs to be relaxed for studying dissociation of OH molecule to O and H. This structure relaxation at the start of Fig. 4d caused a further reduction in the energy compared to the structure shown in 4c. The structural relaxation reduced the magnitude of the out-of-plane deformation of graphene plane and led to an increase of OH distance to the graphene plane at the onset of OH dissociation. As shown in Fig. 4d, the H dissociated from water in Fig. 4c becomes attached to only one dangling carbon atom at monovacancy site. Due to the relaxation process, a discontinuity in the energy (and the distance between O and graphene plane) values between these two figures occurred.

So far, DFT calculations investigated the dissociation of water molecule on PG and MG surfaces and showed that presence of monovacancy would reduce the dissociation energy, resulting in the saturation of the bonds. In multilayer graphene, dissociated OH, H, and O functional groups on each layer interact with the functional groups at the adjacent layer. To analyze the role of dissociated water molecules in modifying the interlayer binding energy between the graphene layers, 6 different types of AB graphene configurations incorporating functional groups with different orientations were considered in following section. It will be shown that the spacing between the graphene layers could increase and layers that were stuck together could separate due to the reduction of interlayer adhesion forces.

### Adhesion and separation of bilayer graphene in presence of dissociated water

**Figure 5:**
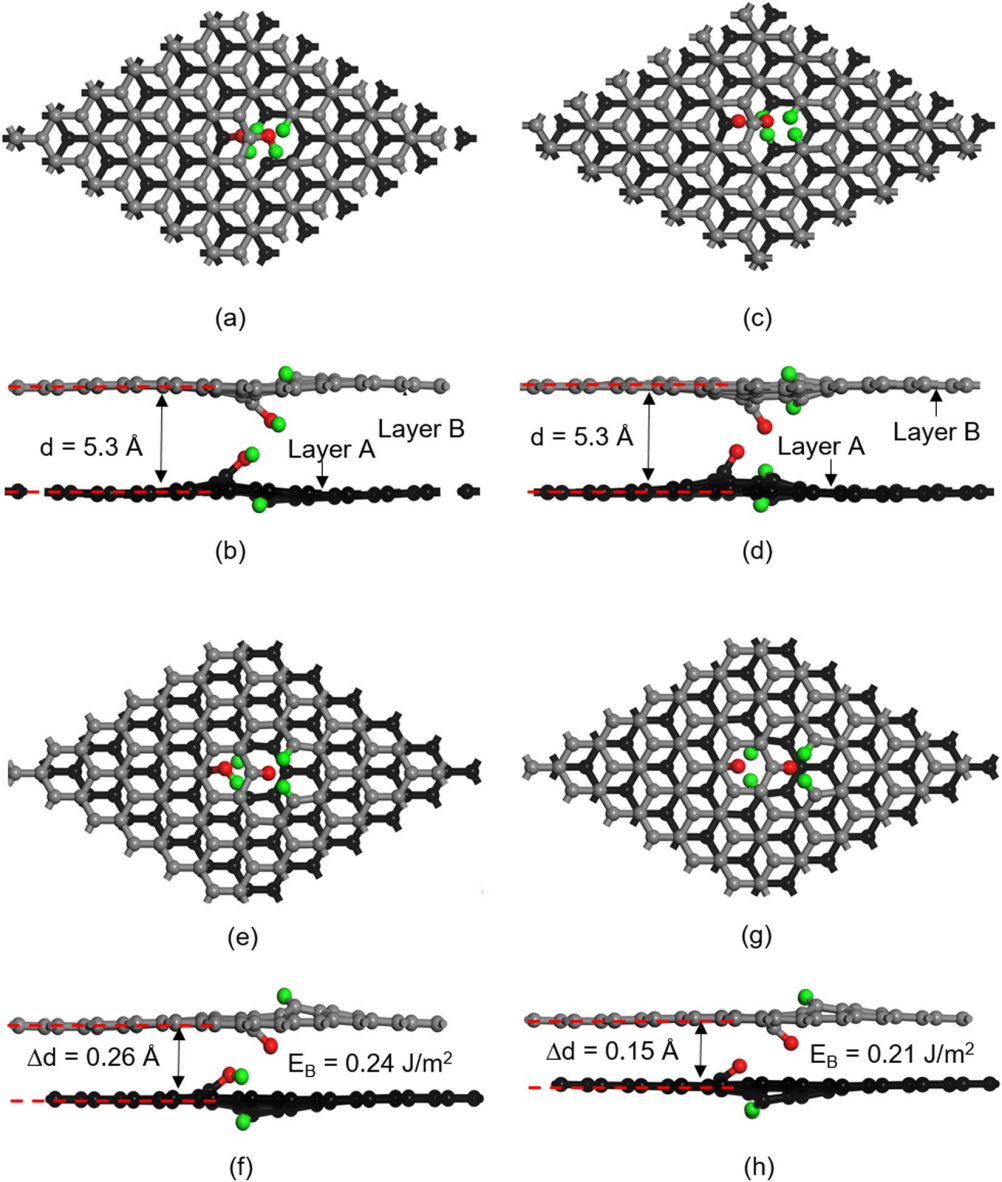
AB type graphene interfaces with the dissociated H_2_O structures showing (**a**) and (**b**) top and side views of H-MG-OH/OH-MG-H; (**c**) and (**d**) top and side views of H-MG-O/O-MG-H. Relaxed structures of AB graphene interfaces showing (**e**) and (**f**) top and side views of H-MG-OH/OH-MG-H; (**g**) and (**h**) top and side views of H-MG-O/O-MG-H. The distortion caused by OH to the attached C atom was 0.7 Å in the Z direction with respect to corner C atom at each layer. C-OH bond length was 1.38 Å. The distortion caused by O atom to the attached C atom was 1.0 Å in the Z direction with respect to corner C atom at each layer. C-O bond length was 1.24 Å.

In order to rationalize the effect of dissociated water molecules on reducing the friction of graphene, the interlayer binding energy of bilayer graphene systems was investigated. The AB type of stacking shown in Fig. 5 is preferable to AA type stacking due to the generation of stronger van der Waals adhesion forces between the AB graphene layers^43^. As described in the previous section, adsorption of the dissociated water molecule occurred at the vacancy site of MG, hence all AB-stacked bilayer graphene configurations were constructed in such a way that each contained a monovacancy (e.g. Fig. 5a). Here, we considered both partial dissociation of H_2_O, namely H and OH adsorption on MG, and full dissociation of H_2_O to 2H and O that were adsorbed on MG. The MG surface with adsorbed OH and H is demarcated as H-MG-OH (see Fig. 5b layer A), and its symmetrical configuration as OH-MG-H on layer B. MG with water fully dissociative adsorbed 2H and O is defined as H-MG-O and its symmetrical configuration as O-MG-H (see Fig. 5(c,d)). In addition to these configurations four others namely, OH-MG-H/H-MG-OH, OH-MG-H/OH-MG-H, O-MG-H/H-MG-O, and O-MG-H/O-MG-H were also considered. Then, all six structures were relaxed to their equilibrium states, and the energy of each layer *E*_*layerA*_ and *E*_*layerB*_ was noted. The interlayer separation between A and B graphene layers in the Z-direction is defined as the average distance between the C atoms at these layers. To simulate the interlayer binding energy between sliding graphene layers with dissociative adsorption of water molecule at defect sites, the functional groups were matched to the nearest distance from the adjacent graphene layer by shifting the layer A shown in Fig. 5(b) and (d) by one C-C bond length in [1, −1, 0] direction. Considering the out-of-plane deformations induced by OH, H, O, the initial separation (*d*_*i*_) between A and B layers was set to 5.3 Å to allow functional groups at both graphene layers to interact with each other. The interlayer binding energy (*E*_*B*_) between the graphene layers (bilayer interfaces) was calculated as ${E}_{B}=({E}_{bilayer}-{E}_{layerA}-{E}_{layerB})/{A}_{i}$, which is the energy difference between the bilayer graphene (${E}_{bilayer}$) and the total energy of the isolated layers per unit interfacial area (${A}_{i}$). The interface energy ${E}_{i}$ was calculated as ${E}_{i}={E}_{total}-{N}_{C}{\mu }_{C}-{N}_{H}{\mu }_{H}-{N}_{O}{\mu }_{O}$, where ${E}_{total}$ is the total energy of the system, *N* is the number of C, H, O atoms, ${\mu }_{C}$ is chemical potential of C atom estimated from the energy of one C atom in bulk graphite, ${\mu }_{O}$ is the chemical potential of O atom estimated as the energy of one O atom from O_2_ in gaseous state (${\mu }_{O}=1/2{E}_{{O}_{2}}$), and ${\mu }_{H}$ is the chemical potential of an H atom estimated as ${\mu }_{H}=1/2({E}_{{H}_{2}O}-{\mu }_{O})$. The results are summarized in Table 1.

**Table 1:** Increase in equilibrium layer spacing ∆d, interlayer binding energy (*E*_*B*_) and interface energy (*E*_*i*_) of AB bilayer graphene with and without dissociated water molecules. The number of graphene unit cells initially covered by water molecules is designated as surface coverage.

Structures	Surface coverage	Increase in layer spacing, ∆d (Å)	Interlayer binding energy *E*_*B*_ (J/m^2^)	Interface energy *E*_*i*_ (J/m^2^)
Pristine graphene	0	0	0.30	0.34
OH-MG-H/H-MG-OH	1/36	0.06	0.28	1.19
O-MG-H/H-MG-O	1/36	0.07	0.27	0.88
H-MG-O/O-MG-H	1/36	0.15	0.21	0.94
H-MG-O/H-MG-O	1/36	0.07	0.27	0.89
H-MG-OH/OH-MG-H	1/36	0.26	0.24	1.09
OH-MG-H/OH-MG-H	1/36	0.10	0.28	1.18
OH-MG-H/OH-MG-H	1/16	0.25	0.26	1.0

According to Table 1 the interlayer separation of the relaxed structures increased when chemisorption of H, OH, and O occurred at the monovacancy site. All graphene interfaces with chemisorbed molecules provided smaller *E*_*B*_ values compared to that of pristine graphene (0.30 J/m^2^); H-MG-O/O-MG-H had the lowest *E*_*B*_ of 0.21 J/m^2^. *E*_*B*_ of the other four graphene interfaces (with the same initial water coverage) were in the range of 0.27~0.28 J/m^2^. The interlayer spacing for H-MG-OH/OH-MG-H configuration with two OH initially facing the interface increased by 0.26 Å (Fig. 5b), resulting in a *E*_*B*_ of 0.24 J/m^2^. The OH at the top graphene layer was further dissociated into O and H, indicating that adjacent graphene layers assisted water molecules to overcome the energy barrier for dissociation. In summary, H-MG-OH/OH-MG-H and H-MG-O/O-MG-H configurations were the ones that had the most significant effect on increasing the interlayer spacing and reducing the interlayer binding energy. The top and side views of H-MG-OH/OH-MG-H structure are shown in Fig. 5(e,f) and those of H-MG-O/O-MG-H in Fig. 5(g,h). It can be noted that the stacking sequence of the relaxed H-MG-OH/OH-MG-H was displaced from the initial AB (see Fig. 5a) towards an AA stacking as shown in Fig. 5(e and f). While the relaxed H-MG-O/O-MG-H structure preserved the initial AB stacking as shown in Fig. 5(g and h), the top layer (layer B) was shifted by two C-C bond length in [1, −1, 0] direction with respect to the lower layer (layer A).

Another important piece of information that arises from the data shown in Table 1 is that increasing the initial water coverage on graphene surfaces (from 1/36 to 1/16 in OH-MG-H/OH-MG-H) would result in an increase in the interlayer spacing and a reduction in the interlayer binding energy. An examination of the interface energy values of the bilayer graphene in Table 1 indicates that the graphene structures incorporating fully dissociated water (H-MG-O) are more energetically favourable than the H-MG-OH. This can be seen by comparing interface energies of O-MG-H/H-MG-O (0.88 J/m^2^), H-MG-O/H-MG-O (0.89 J/m^2^) and H-MG-O/O-MG-H (0.94 J/m^2^). As the interface energies for all configurations are essentially similar (0.9–1.2 J/m^2^), it is conceivable that all bilayer graphene configurations considered may form concurrently and coexist.

### Friction reduction mechanisms of graphene

One of the salient points arising from the results presented above was that graphene showed the lowest running-in COF compared to the other carbon based materials namely, DLC and PCD tested against the same metallic counterface. Formation of carbonaceous transfer layers on the counterface was a common characteristic feature of all the materials tested. The results also revealed that achievement of a low steady-state COF was possible when the graphene was tested in humid atmospheres. Sliding contact under an inert atmosphere would create defects with a high µ_R_ but would not lend itself to a steady state friction regime. Raman spectra of the graphene wear track revealed the occurrence of a D peak at 1330 cm^−1^, which infers that defects were generated during sliding. It could be germane to comment here that graphene has a distinctly different Raman fingerprint that differentiates it from graphite as indicated in the introduction section. Pristine graphene is hydrophobic but a graphite structure consists of dangling carbon atoms. Dangling carbon atoms on the graphite surfaces have a tendency to promote dissociative adsorption of water molecules^5,6^. Creation of sliding induced defects is an essential step for friction reduction in graphene as demonstrated in this paper. First principles calculations suggested that adsorption of H, O, and OH groups that were dissociated from water molecules in the environment during sliding would reduce the interlayer binding energy between the adjacent graphene layers. The formation of C-O, C-H, and C-OH bonds could lead to distortion of the graphene planes, which may further facilitate dissociative adsorption of water molecules on the graphene surfaces. The sliding induced defects were important for the dissociation process as they reduced the energy required for this process compared to pristine graphene prior to the sliding. If the defect sites were not passivated the COF would increase as illustrated by the experiment carried on the graphene sliding under a nitrogen atmosphere (Fig. 3). According to the results of first principles studies the interplanar spacing of graphene layers increased as a result of adsorption of dissociated water molecules (see Fig. 5(f,h) and Table 1). An increase in the spacing of the graphene layers subjected to sliding contact damage was observed experimentally as discussed below.

**Figure 6:**
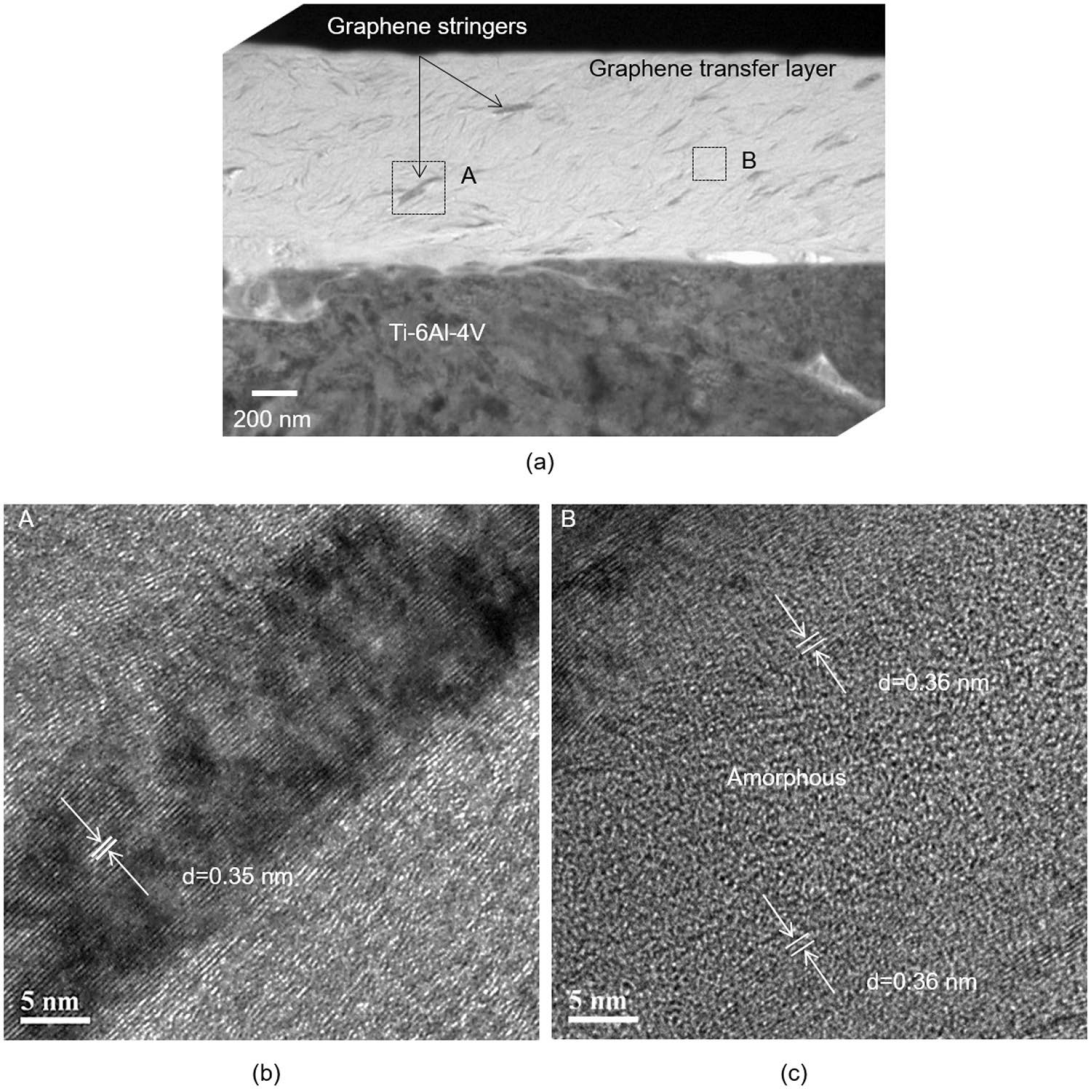
(**a**) Cross-sectional TEM image of the transfer layer formed on the counterface placed in sliding contact with graphene; (**b**) high resolution TEM (HR-TEM) image acquired from the region indicated as (A) in (**a**) showing graphene stacks with an average d-spacing of 0.35 nm; (**c**) HR-TEM image acquired from the region indicated as (B) in (**a**) showing graphene stacks (with d-spacing 0.36 nm) embedded in an amorphous matrix.

**Figure 7:**
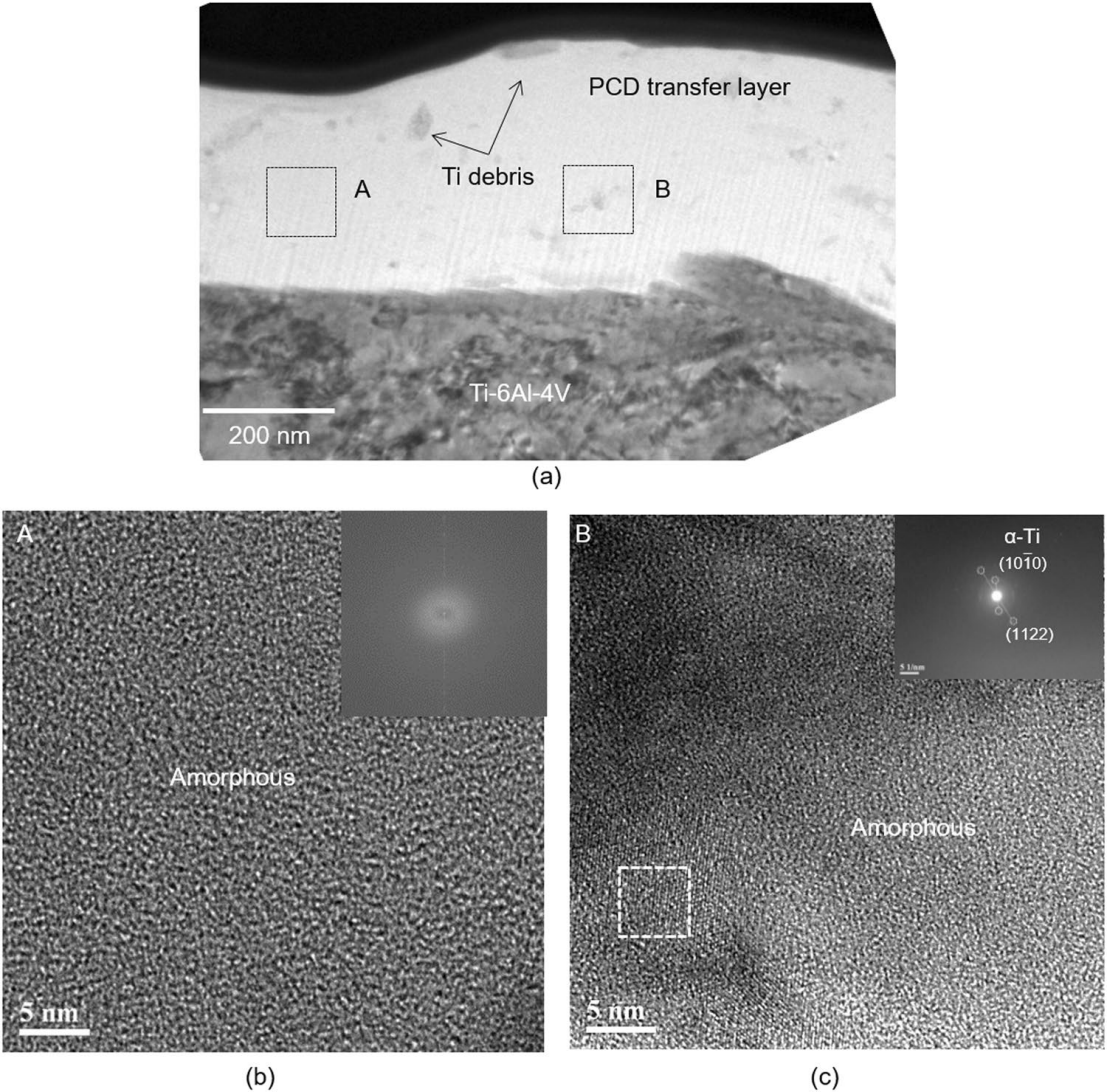
(**a**) Cross-sectional TEM image of the transfer layer formed on the counterface placed in sliding contact with PCD; (**b**) high resolution TEM (HR-TEM) image acquired from the region indicated as (A) in (**a**) showing an amorphous C matrix. The inset of (**b**) shows the Fast Fourier Transform (FFT) derived diffraction patterns of transfer layer; (**c**) HR-TEM image acquired from the region indicated as (B) in (**a**) showing an amorphous C matrix with α-Ti. FFT derived diffraction pattern of α-Ti is shown in inset.

Cross-sectional TEM investigations conducted on the transfer layers formed on the counterface indicated that the layers incorporated of graphene ‘folds’ with an average length of 133 ± 49 nm, and a width of 12 ± 6 nm (Fig. 6a). High resolution TEM (HR-TEM) images shown in Fig. 6(b) identified that these folds consisted of a few layers of graphene stacked adjacent to each other and embedded in an amorphous carbon matrix (Fig. 6c). The d-spacing between of the graphene layers in these stacks were >0.34 nm according to the Fast Fourier Transform (FFT) derived diffraction patterns taken from samples shown in Fig. 6(b and c). The most significant feature of the HR-TEM studies was therefore the observation that the d-spacing values of the graphene stringers in the transfer layers were larger than that of pristine graphite with d_(002)_ = 0.334 nm^44^. In contrast, the transfer layer that was generated under a N_2_ atmosphere was amorphized during sliding. No remnants of graphene stacks layers could be detected (see Supplementary Fig. S1). Thus, the damage was more severe and a complete amorphization appeared to have been occurred when the graphene was tested in a nitrogen atmosphere. The COF was high as shown in Fig. 3. A cross-sectional view of transfer layers generated during sliding of PCD is shown in Fig. 7(a). The transfer layer had an amorphous structure with occasional fragments of titanium debris (with an average length of 820 ± 10 nm and width of 15 ± 7 nm) embedded in the amorphous matrix as shown in Fig. 7(b,c), indicating that the transfer of metallic debris particles would occur during the running-in period of sliding of PCD. This was accompanied by a high friction during the running-in period which was also longer in duration compared to that of the graphene. Similarly, the DLC coatings tested had metallic fragments incorporated in the transfer layers formed on the counterfaces run against them. The transfer layers generated during sliding of graphene did not indicate formation of metallic debris.

As stated earlier, dissociation of water at a monovacancy site of graphene showed a lower *E*_*b*_ of 1.27 eV compared to that of PG (3.53 eV), and the *E*_*b*_ of graphene could be further lowered due to the generation of defects during sliding. This could be attributed to the adsorption of H_2_O and OH at the vacancy sites on thegraphene planes. In contrast, water can be easily dissociated on cleaved diamond (111) surface with a low *E*_*b*_ of 0.41 eV, indicating freshly cleaved diamond surface is more reactive than a monovacancy graphene surface. However, as the amorphous DLC and the PCD would need to be graphitized before being transferred to the counterface and the magnitudes of µ_R_ of DLC and PCD were higher and the duration of the running in-period were longer than that observed for graphene. Once the transfer layer was formed and the graphene planes were passivated by the dissociated water molecules at sliding induced defect sites on graphene surface, a steady state friction regime was established.

In summary, sliding induced defects generated on the contact surfaces of graphene in ambient air act favourably and assist with the formation of transfer layers. As a result a steady state friction regime with a low µ_S_ would occur–a process that requires dissociative adoption of water molecules. The interpretations of the proposed friction reduction mechanisms are well supported by the experimental observations as well as by the results of the first principles calculations. However, the details of generation of sliding induced defects should be further studied.

## Conclusions

Sliding contact experiments and DFT calculations that used van der Waals interactions elucidated the friction reduction mechanisms of graphene by determining the roles of structural defects and dissociated water molecules. The sliding friction tests showed low steady state COF values in ambient and humid atmospheres but not in an inert atmosphere. Cross-sectional TEM studies revealed that a tribolayer was formed on the counterface and consisted of an amorphous carbon matrix with intermittent stringers of graphene stacks; the d-spacing between the graphene layers was larger compared to that of the pristine graphene. On the other hand, the transfer layer formed from PCD exhibited an amorphous carbon matrix incorporating metallic debris as inclusions.

DFT calculations showed that compared to pristine graphene, water was more likely to be dissociatively adsorbed on graphene incorporating a monovacancy defect due to a lower energy barrier of water dissociation into OH, H and O. the dissociated moelcules served to passivate the dangling carbon bonds. Dissociated water molecules intercalated between the graphene layers increased the graphene layer spacing as observed experimentally, and reduced the interlayer binding energy by ~30% compared to pristine graphene. In comparison, dissociated water molecules was adsorbed only at the surface C atoms PCD (and DLC). The H, O, and OH intercalation between the graphene layers allowed the graphene layers to be transferred readily to the counterface resulting in a lower COF running-in period with shorter duration compared to those of other carbon based materials, namely DLC and PCD.

## Methods

### Description of samples and tribological experiments

Sliding friction tests on graphene were performed against both Ti-6Al-4V and H-DLC coated tool steel. Graphene samples were deposited on Ni foils using a chemical vapour deposition (CVD) process. Methane gas was utilized as the source of carbon. Micro-Raman spectra obtained using a Horiba Raman micro-spectrometer with 50 mW Nd–YAG laser (532 nm excitation line) indicated a single sharp 2D peak at ∼2700 cm^−1^. The ratio of G peak at 1580 cm^−1^ to 2D peak was equal or greater than one in many locations indicating a multi layered structure^25,45^. There was no evidence for the formation of a D peak at 1330 cm^−1^ suggesting that the defect density of the pristine multilayer graphene plates was low. Sliding friction tests were also conducted on polycrystalline (35° rhombic) diamond, PCD and diamond-like carbon (DLC) coatings. The DLC coatings were deposited using an unbalanced magnetron sputtering system that used one chromium (to deposit an interlayer) and two graphite targets. Butane precursor gas was used to produce H-DLC type coating with 40 at.% hydrogen measured by elastic recoil detector (ERD). The hydrogen content of the NH-DLC type coating was <2 at.%.

A pin-on-disk tribometer was used to measure the COF of multilayered graphene samples and other carbon surfaces against Ti-6Al-4V alloy pins with hemispherical tips of 4.05 mm in diameter. A constant speed of 0.05 m/s and a normal load of 1.00N were applied for 500 revolutions. Sliding tests were conducted under the ambient atmospheric conditions with 22% RH. A particular focus of the work was to understand of the formation of sliding induced structural defects as well as to determine the origin of the tribolayers generated on the contact surfaces. For this purpose, special emphasis was placed on studying the running-in behaviour of graphene in the initial stage of friction where the tribolayers were first generated. The highest value of COF reached during the running-in stage and the duration of running-in stage were noted. The steady state COF, µ_S_, values were calculated from the arithmetic mean of the COF signals of each curve following the running in stage. Each set of tests on a given type of sample was repeated three times. The average values of µ_R_ and µ_S_ were calculated. Tests conducted using H-DLC counterfaces in a dry N_2_ atmosphere are described in Results and Discussion section.

A FEI Quanta 200 FEG scanning electron microscope (SEM) equipped with an energy-dispersive X-ray EDAX SiLi Detector spectrometer was used to study the contact surfaces. Cross-sectional transmission electron microscopy (TEM) samples taken from the transfer layers were excised using a focused ion beam (FIB) lift out technique in a Carl Zeiss NVision 40 dual beam work station. The samples were ion-milled from both sides to a thickness of about 100 nm. The final milling of the samples was conducted at a low beam current of 40 pA. TEM observations were performed using FEI Titan 80–300 HR-TEM, with a lateral resolution <1 Å, operated at 300 kV.

### First principles calculations

These calculations were performed using the projector-augmented wave (PAW) method with the exchange correlations that incorporated van der Waals interactions in the framework of vdW-DF2 functional^37,46^ as implemented in the Vienna Ab Initio Simulation Package (VASP)^47,48^. The generalized gradient approximation (GGA)^49^ of the exchange correlation described by Perdew, Burke, and Ernzerhof (PBE)^50^ was used with a plane-wave cut off energy of 500 eV to reach the energy convergence of 1–2 meV/atom. The exchange-correlation energy ${E}_{XC}$ in the non-local van der Waals density functional is described as: ${E}_{XC}={E}_{X}^{GGA}+{E}_{C}^{LDA}+{E}_{C}^{nl}$ ^51^, where ${E}_{X}^{GGA}$ is the GGA exchange energy, ${E}_{C}^{LDA}$ is the local correlation energy obtained within the local density approximation (LDA), and ${E}_{C}^{nl}$ is the non-local correlation energy. To calculate ${E}_{X}^{GGA}$, rPW86^37^ exchange functional was used. The electronic degrees of freedom were converged to 10^−5^ eV/cell, and the Hellman-Feynman forces were relaxed to less than 0.05 eV/Å via the conjugate gradient method. A 24 × 24 × 10 Monkhorst-Pack grid of k-points was used for unit cell of graphite, whereas a grid of 4 × 4 × 1 k-points was used for all water/(6 × 6)graphene slabs. For diamond, 20 × 20 × 20 k-points mesh was used while a mesh of 8 × 8 × 1 was sufficient to converge the energies of water/(3 × 3)diamond (111) slabs. Spin polarized calculations were performed to account for the magnetism incurred by vacancy formation in graphene^52,53^, and were employed for all applicable simulation systems.

Prior to using vdW-DF2 for calculating the thermodynamic and structural properties of multilayered graphene, lattice constants and binding energy between graphite layers were calculated. We obtained the lattice constants of bulk (1 × 1) graphite *a* = 2.48 Å, and interlayer separation is *c* = 3.518 Å. The calculated interlayer binding energy per carbon atom of 52.6 meV/atom was consistent with 52.0 meV/atom calculated using the vdW-DF2 method^54^, and agreed well with the experimental value of 52 meV/atom^55^, although the vdW-DF2 slightly overestimated the interlayer separation by about 6% compared with the experimental value of 3.34 Å^56^. The lattice constant obtained for diamond, *a* = 3.61 Å, was the same as that reported in the literature calculated by vdw-DF2, which was 2% higher than the experiment value of 3.54 Å^57^.

**Figure 8:**
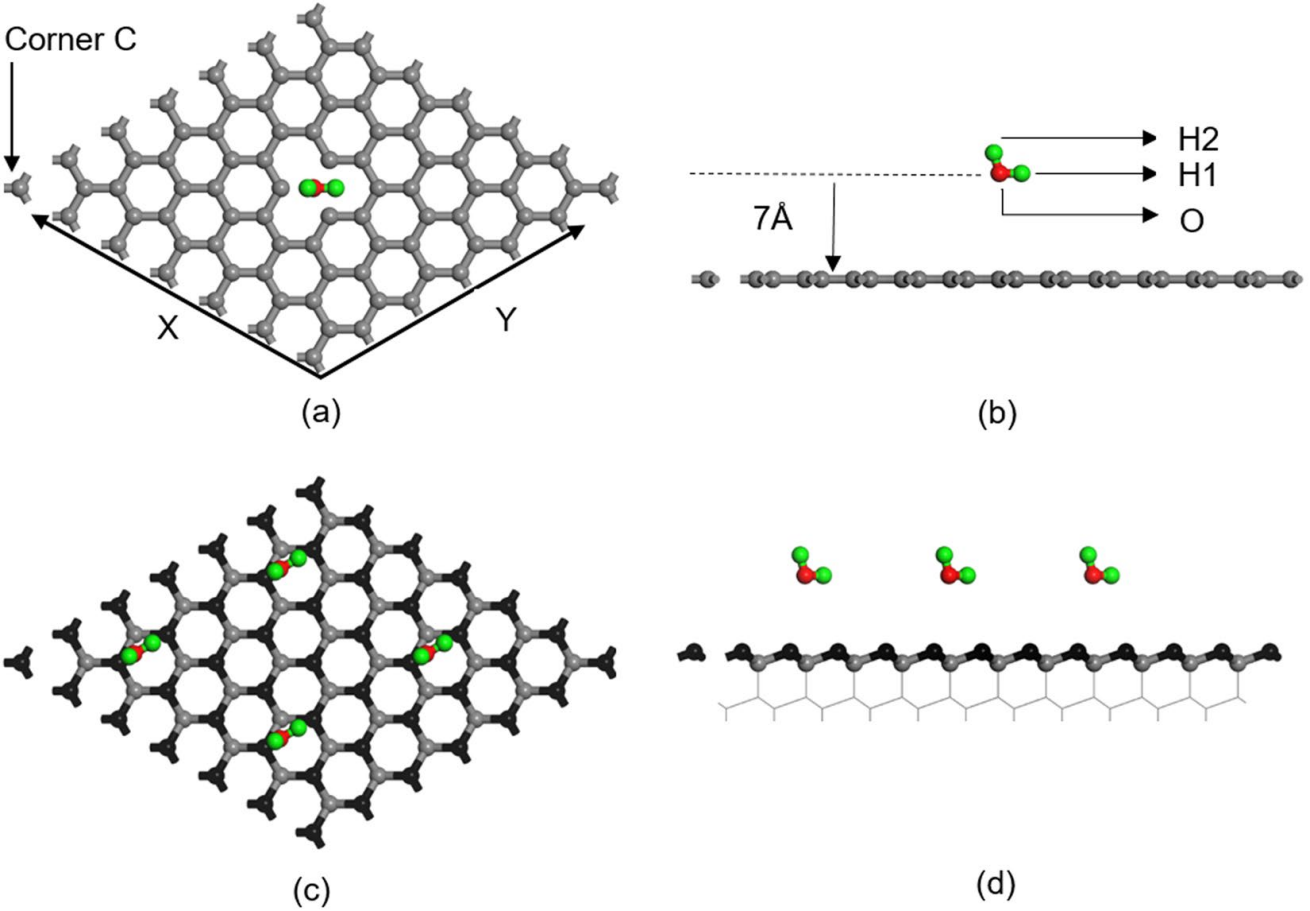
(**a**) Plane view of graphene (6 × 6 cell) with monovacancy and an approaching H_2_O molecule. The O atom of O-H bond was located where the C atom was removed in order to create the monovacancy; (**b**) Side view of an approaching H_2_O molecule to a graphene with monovacancy; (**c**) Plane view of diamond (6 × 6 cell) and a H_2_O molecule approaching diamond (111) surface. The O atom and H atom of O-H bond was located at the bridge site of two elevated carbon atoms (black); (**d**) Side view of H_2_O molecules approaching the bridge sites on diamond (111) surface.

The computed O-H bond length, 0.971 Å, of the H_2_O molecule and the bond angle of 104.8° (by relaxing the water molecule in a cubic box with 10 Å cell edge) agreed with the experimental bond length value of 0.958 Å and bond angle of 104.8°^58^, and these values were used in the simulation of the reaction mechanisms between water and the graphene. The reaction of water with graphene with monovacancy was simulated by gradually bringing a water molecule from a large separation distance (7 Å) towards the vacancy site on (6 × 6) graphene plane. A vacancy was created by removing one C atom at the center of (6 × 6) graphene cell (with 71 C atoms) as shown in Fig. 8(a). The initial water orientation was carefully selected in order to achieve a low water dissociation energy barrier configuration; Accordingly, H1 and O atoms in the initial water molecule were placed at the closest locations to each of the three dangling carbon atoms surrounding a monovacancy site. Therefore, as shown in Fig. 8(a), the water molecule on graphene was located such that O-H1 was parallel to the graphene surface with its centre positioned at the centre of vacancy site and O-H2 formed an angle of 14.8° from the centre of the vacancy and the Z axis. The center of O-H1 bond was considered to coincide with the centre of the monovacancy as this configuration would minimize energy expenditure due to water molecule reorientation before dissociation. This method is in agreement with previous water dissociation calculations on graphene with monovacancy^30^. The same water orientation with O and H1 located at the center of a carbon hexagonal ring of the (6 × 6) pristine graphene with O-H2 making the same angle of 14.8° in the Z direction was maintained. Therefore, the initial configurations of water molecule in the pristine graphene and graphene with monovacancy were the same. Consequently, the energy barrier calculations for physisorption and water dissociative adsorption of water molecule on pristine (Fig. 4b) and monovacancy graphene (Fig. 4c and d) are comparable.

A (6 × 6) graphene cell (with 72 C atoms) was selected to accommodate possible deformation of graphene in the normal direction (Z direction) in Fig. 8(b). The graphene cell size was large enough to minimize the interactions between the periodic images of vacancies. A 15 Å vacuum layer was added between the graphene layers and the resulting cell was large enough to prevent the interactions between the molecules in the image cells. One water molecule on (6 × 6) graphene occupied a surface area corresponding to 1/36^th^ of the monolayer. The distance between an approaching water molecule and the graphene plane was defined from the distance between the location of the O atom and the corner C atom of graphene in the Z-direction. During simulation of the change in the energy at a given separation distance for a water molecule approaching from an initial distance of 7 Å until dissociation, the Z-coordinates of O and C atoms were fixed in the Z-direction, while all the other atomic coordinates were fully relaxed.

For studying water reaction with diamond, a cell of four double-layers of diamond (111) surface with (3 × 3) cell size and dimension of 7.65 Å × 7.65 Å × 21.78 Å was used to simulate bulk diamond by fixing the bottom two double-layers of carbon atoms in all directions. Here, the reconstructed diamond (111)−2 × 1 surface (Pandey structure) was not used because this structure provided a high *E*_*b*_ for water dissociation. A 15 Å vacuum layer was added between the carbon layers. The plane view of O-H1 of a H_2_O molecule at bridge site of diamond (111) surface and the side view of a water molecule approaching to diamond surface are shown in Fig. 8(c) and (d), respectively. O-H1 was initially at the bridge site of the two nearest carbon atoms at top carbon layer, and 7 Å above the diamond (111) plane as shown in Fig. 8(c,d), and this molecule was allowed to approach towards diamond (111) plane until the O-H1 bond was broken. O and H1 were also fixed in Z direction while all the other atomic coordinates were fully relaxed. The distance between an approaching water molecule and the diamond surface was defined from the locations of the O atom and the C atom of diamond in the Z-direction.

## Electronic supplementary material

Supplementary Information
