# Supplementary material for: Roles of sliding-induced defects and dissociated water molecules on low friction of graphene
Source: Sci Rep. 2018 Jan 9;8:121. doi: 10.1038/s41598-017-17971-1 (PMC5760666; doi:10.1038/s41598-017-17971-1)
Supplement: Supplementary file 1 — Supplementary Information [file 41598_2017_17971_MOESM1_ESM.pdf]

Supplementary Information

Roles of sliding-induced defects and dissociated water molecules on low friction of graphene

Zaixiu Yang[1], Sukanta Bhowmick[1], Fatih G Sen[2], Anindya Banerji[1], Ahmet T Alpas[1]*

[1]*Engineering Materials Program, Mechanical, Automotive and Materials Engineering Department, University of Windsor, Windsor, ON N9B3P4, Canada*

[2]*Center for Nanoscale Materials, Argonne National Laboratory, Cass Ave., Lemont, IL 60439, USA*

*Corresponding author: A. T. Alpas (email: aalpas@uwindsor.ca)

Supplementary Figure S1

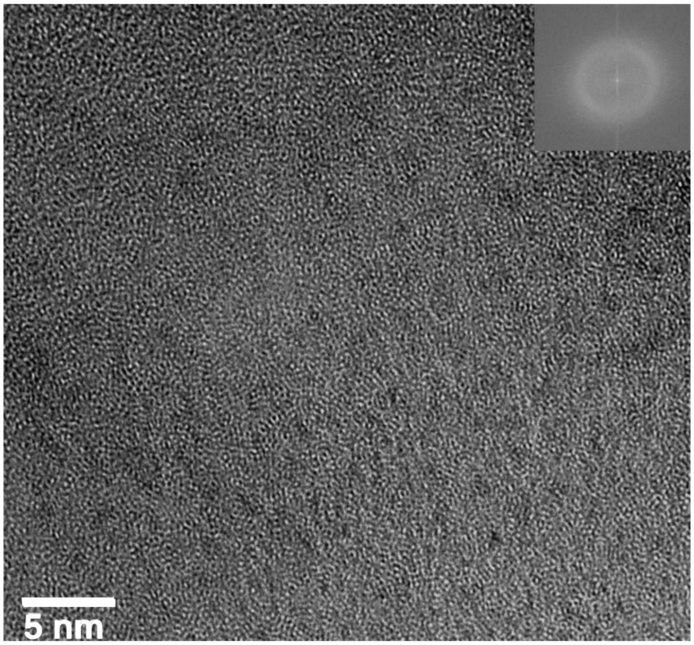

Supplementary Figure S1. Cross-sectional high resolution TEM image of the transfer layer formed on the counterface after sliding of graphene under a dry N_2 atmosphere (<4% RH) using a normal load of 1.00 N and a speed of 0.05 m/s for 1000 revolutions indicating presence of an amorphous structure (see the inset).
